# Oxidative stress-mediated mitochondrial dysfunction facilitates mesenchymal stem cell senescence in ankylosing spondylitis

**DOI:** 10.1038/s41419-020-02993-x

**Published:** 2020-09-17

**Authors:** Guiwen Ye, Zhongyu Xie, Huiqiong Zeng, Peng Wang, Jinteng Li, Guan Zheng, Shan Wang, Qian Cao, Ming Li, Wenjie Liu, Shuizhong Cen, Zhaofeng Li, Yanfeng Wu, Zhizhong Ye, Huiyong Shen

**Affiliations:** 1 Department of Orthopedics, Sun Yat-Sen Memorial Hospital, Sun Yat-Sen University, Guangzhou, 510120 P.R. China; 2 Department of Orthopedics, The Eighth Affiliated Hospital, Sun Yat-Sen University, Shenzhen, 518033 P.R. China; 3 Shenzhen Futian Hospital for Rheumatic Diseases, Shenzhen, 518040 P.R. China; 4 Center for Biotherapy, The Eighth Affiliated Hospital, Sun Yat-Sen University, Shenzhen, 518033 P.R. China; 5 Center for Biotherapy, Sun Yat-Sen Memorial Hospital, Sun Yat-Sen University, Guangzhou, 510120 P.R. China

**Keywords:** Senescence, Mesenchymal stem cells, Ankylosing spondylitis

## Abstract

Ankylosing spondylitis (AS) is a chronic inflammatory disease possessing a morbid serum microenvironment with enhanced oxidative stress. Long-term exposure to an oxidative environment usually results in cellular senescence alone with cellular dysfunction. Mesenchymal stem cells (MSCs) are a kind of stem cell possessing strong capabilities for immunoregulation, and senescent MSCs may increase inflammation and participate in AS pathogenesis. The objective of this study was to explore whether and how the oxidative serum environment of AS induces MSC senescence. Here, we found that AS serum facilitated senescence of MSCs in vitro, and articular tissues from AS patients exhibited higher expression levels of the cell cycle arrest-related proteins p53, p21 and p16. Importantly, the levels of advanced oxidative protein products (AOPPs), markers of oxidative stress, were increased in AS serum and positively correlated with the extent of MSC senescence induced by AS serum. Furthermore, MSCs cultured with AS serum showed decreased mitochondrial membrane potential and ATP production together with a reduced oxygen consumption rate. Finally, we discovered that AS serum-induced mitochondrial dysfunction resulted in elevated reactive oxygen species (ROS) in MSCs, and ROS inhibition successfully rescued MSCs from senescence. In conclusion, our data demonstrated that the oxidative serum environment of AS facilitated MSC senescence through inducing mitochondrial dysfunction and excessive ROS production. These results may help elucidate the pathogenesis of AS and provide potential targets for AS treatment.

## Introduction

Ankylosing spondylitis (AS) is a chronic inflammatory disease with high morbidity and disability rates^1^. Chronic inflammation is a core feature of AS pathogenesis, but the associated mechanism is still unclear^2^. Alone with the chronic inflammation, AS patients always maintain a morbid serum microenvironment, which has been reported to be abnormal in terms of inflammatory factor and oxidative stress levels^3,4^. Previously, we also found that the levels of advanced oxidative protein products (AOPPs), a marker of oxidative stress, were elevated in AS and positively correlated with disease activity^5^. However, whether and how the AS serum microenvironment participates in AS pathogenesis are still unknown.

Mesenchymal stem cells (MSCs) are a kind of stem cell that possesses strong capabilities for immunoregulation and trilineage differentiation^6^. MSCs dysfunction is tightly involved in multiple inflammatory diseases, such as osteoarthritis (OA), rheumatoid arthritis (RA), and AS^7,8^. Previous studies discovered that MSCs from AS patients exhibited multifunction disorder and MSCs from healthy donors (HDs) were effective for treating AS^9–12^, indicating a crucial role for MSCs dysfunction in AS pathogenesis. Thus, whether the morbid serum microenvironment of AS influences the functions of MSCs remains to be addressed. No relevant studies have been reported.

Cellular senescence is a cellular status characterized by irreversible cell cycle arrest in G0/G1, increased expression of cell cycle arrest-related proteins and high activity of senescence-associated β-galactosidase (SA-β-gal)^13^. This state is usually induced by multiple replication and environmental stresses, such as an inflammatory condition or oxidative stress^14^. In addition, senescence always accompanies cellular dysfunction and may be involved in pathological processes^15,16^. It has been reported that senescent MSCs display a senescence-associated secretory phenotype (SASP), an impaired immunomodulatory function and aberrant differentiation capability^17,18^. Furthermore, in some autoimmune diseases such as systemic lupus erythematosus (SLE)^19^ and systemic sclerosis (SSc)^20^, MSCs senescence is induced by the patient’s serum and participates in disease development. However, whether a similar situation exists in AS remains unclear.

In this study, we determined the effect of the AS serum microenvironment on MSCs senescence and demonstrated that compared to serum from HDs, AS serum strikingly facilitated the senescence of MSCs. We further explored the underlying mechanism, and our data illustrated that the AOPPs in AS serum and the downstream mitochondrial dysfunction and excess ROS expression of MSCs were attributed to the senescence-promoting effect of the AS serum. Our findings suggest a role for the AS serum microenvironment in AS pathogenesis and provide several new targets for AS therapy.

## Materials and methods

### Ethical statement

This study was approved by the Ethics Committee of Sun Yat-Sen Memorial Hospital, Sun Yat-Sen University, Guangzhou, China. Written informed consent about the experimental requirements and potential risks was provided by all subjects.

### Study subjects

In this study, the population consisted of 20 HDs and 20 AS patients. All the AS patients fulfilled the modified New York criteria^21^ and were in an active disease state (Bath Ankylosing Spondylitis Disease Activity Index (BASDAI) ≥ 4)^22^. The characteristics of the study subjects are presented in Supplemental Table 1.

### Isolation and culture of MSCs

HDs were recruited, and MSCs were isolated and cultured as described previously^23^. Briefly, bone marrow puncture was performed, and MSCs were isolated and purified immediately by density gradient centrifugation at 2500*g* for 30 min. Then, the MSCs were resuspended in Dulbecco’s modified Eagle’s medium (Gibco, 11885-076) containing 10% fetal bovine serum (Zhejiang Tianhang Biotechnology, 11011-8611) and seeded in culture flasks. After the cells were cultured in incubators at 37 °C with 5% CO_2_ for 2 days, the medium was replaced to remove the cells in suspension. Subsequently, the culture medium was replaced every 3 days. In addition, when the cells reached 80–90% confluence, they were digested by using 0.25% trypsin containing 0.53 mM ethylenediamine tetraacetic acid (EDTA) and reseeded in two new culture flasks. In addition, the MSCs were used for the following experiments at passage 4.

### Serum extraction and usage

Peripheral blood from the HDs and AS patients was collected in EDTA anticoagulant tubes. Then, the blood was centrifuged at 2000*g* for 15 min, and heat-inactivation was performed by heating the samples at 56 °C for 30 min. Then, the serum was stored at −80 °C until use, and 10% serum was used for MSCs culture.

### SA-β-Gal staining

The Senescence-Associated β-gal Assay Kit was purchased from Beyotime Biotechnology (C0602), and SA-β-gal staining was conducted according to the manufacturer’s instructions. Briefly, MSCs were seeded in 6-well plates and cultured with HD serum or AS serum for 72 h. Then, the cells were washed with PBS and fixed with 1 ml of fixing solution per well. After being incubated at room temperature for 15 min, the cells were washed twice with PBS and incubated with a freshly prepared SA-β-gal staining solution in an incubator at 37 °C with low CO_2_ for 16–18 h. Senescent cells were observed under a light microscope and evaluated by counting the number of blue-stained cells. For tissue slice staining, frozen sections were fixed with fixing solution for 15 min and stained with SA-β-gal staining solution in an incubator at 37 °C with low CO_2_ for 16–18 h. Then, the sections were counterstained with eosin and observed under a light microscope.

### Western blotting

The relevant protocols were reported previously^10^. Briefly, MSCs were cultured with HD serum or AS serum for 2 days and lysed with RIPA buffer containing protease and phosphatase inhibitors for 30 min on ice. The lysates were collected and centrifuged at 14,000 rpm for 30 min at 4 °C. The protein concentrations of the lysates were detected with the BCA Protein Assay Kit (CWBIO, CW0014S), and equal amounts of proteins were mixed with a 5× sodium dodecyl sulfate (SDS) loading buffer. Then, the proteins were separated via SDS-polyacrylamide gel electrophoresis and transferred to polyvinylidene fluoride membranes (Millipore, IPVH0010). After being blocked with 5% non-fat milk for 1 h, the membranes were incubated with primary antibodies against p53 (1:1000, Cell Signaling Technology, 2524S), p21 (1:1000, Cell Signaling Technology, 2947S), p16 (1:1000, Cell Signaling Technology, 80772S) or GAPDH (1:2000, CWBIO, CW0100M) at 4 °C overnight. Then, the membranes were incubated with HRP-conjugated secondary antibodies (1:3000, Boster, BA1050 and BA1054) for 1 h and detected by using Immobilon Western Chemiluminescent HRP Substrate (Millipore WBKLS0500).

### Frozen tissue section preparation

Fresh articular soft tissue samples were collected from four AS patients and four non-AS patients (patients with osteonecrosis of the femoral head) during surgery. Then, the tissue samples were embedded in optimal cutting temperature (OCT) compound and frozen with liquid nitrogen immediately. The frozen tissue samples were sectioned with a freezing microtome, and the sections were stored at −80 °C until use.

### Immunofluorescence

MSCs seeded on coverslips or frozen sections of the above described tissue samples were fixed with paraformaldehyde for 15 min and subsequently permeabilized with 1% Triton X-100 for 20 min. Then, the slides were blocked with 10% goat serum for 30 min and incubated with anti-p-H2A.X (1:400, Cell Signaling Technology, 9718T), anti-CD105 (1:500, Abcam, ab231774), anti-p53 (1:4,000, Cell Signaling Technology, 2524S), anti-p21 (1:250, Invitrogen, 33-7000), or anti-p16 (1: 1000, Abcam, ab16123) antibodies at 4 °C overnight. Then, the slides were stained with fluorophore-labeled secondary antibodies (1:500, Cell Signaling Technology, 4409S and 4412S), and 4′,6-diamidino-2-phenylindole (DAPI) was used to stain cell nuclei. The slides were observed with a laser scanning confocal microscope (Nikon Eclipse Ni-E) or a fluorescence microscope according to the manufacturer’s instructions.

### Cytokine array assay

The Proteome Profiler Human XL Cytokine Array Kit (R&D Systems, ARY022B) was used according to the manufacturer’s instructions. The sera obtained from two AS patients and two HDs were used, and cytokine optical densities were quantified using HLImage++ software (Western Vision).

### Enzyme-linked immunosorbent assay (ELISA)

The serum concentrations of CHI3L1 and PDGFA were detected using Quantikine ELISA kits for human CHI3L1 and PDGFA, respectively (R&D Systems, DC3L10 and DY221), according to the manufacturer’s instructions. The absorbances of the reactants were measured using a Varioskan Flash Multimode Reader (Thermo Fisher), and the concentrations were determined according to the standard curves generated using the standard substance.

### AOPP detection

The detection procedure was performed as described previously^24^. The concentration of AOPPs in serum was assessed using spectrophotometry and is expressed in equivalents of chloramine-T. In brief, 40 µl of serum diluted with 160 µl of PBS, 200 µl of chloramine-T (for calibration, Sigma, St. Louis, MO, USA) and 200 µl of PBS (blank control) were added to a 96-well plate. Then, 20 µl of acetic acid and 10 µl of 1.16 M potassium iodide were added to each well, and the absorbance at 340 nm was tested immediately. The concentrations of serum AOPPs are expressed as µmol/L of chloramine-T equivalents.

### ROS detection

Total ROS levels were measured with the Reactive Oxygen Species Assay Kit (Beyotime, China, S0033), which is based on the fluorescent probe DCFH-DA. Briefly, after being cultured with HD serum or AS serum for 2 days, MSCs were collected and incubated with 10 µmol DCFH-DA for 20 min at 37 °C while protected from light. Then, the cells were washed three times with serum-free medium, and the fluorescence of DCF (excitation: 488 nm, emission: 530 nm) was determined with a flow cytometer (BD Influx, BD Bioscience) and a fluorescence microscope. ROS levels are expressed as the mean fluorescence intensity (MFI) of DCF.

### Mitochondrial membrane potential detection

Mitochondrial membrane potential (∆Ψm) was detected with the ∆Ψm Kit of JC-1 (Beyotime, China, C2006) according to the manufacturer’s instruction. At a high ∆Ψm, JC-1 probes gather together and emit red fluorescence, while at a low ∆Ψm, they remain monomers and emit green fluorescence. After being cultured with HD serum or AS serum for 2 days, MSCs were incubated and washed according to the kit protocol and then detected under a fluorescence microscope. The ∆Ψm is expressed as the fluorescence intensity ratio of red/green analyzed by ImageJ.

### ATP detection

The ATP Assay Kit basing on firefly luciferase (Beyotime, China, S0026) was applied to determine cellular ATP levels according to the manufacturer’s protocol. Briefly, MSCs were cultured with HD serum or AS serum for 2 days. Then, proteins were extracted, and the protein concentrations were measured according to the methods mentioned above. The ATP levels were detected with a luminometer and are expressed as nmol/mg of protein.

### Mitochondrial OCR detection

The mitochondrial oxygen consumption rate (OCR) was determined by using the Seahorse XF Cell Mito Stress Test Kit (Seahorse, 103015-100) and Seahorse XF Cell Mito Stress Test Starter Pack (Seahorse, 102601-100) according to the manufacturer’s instructions. Briefly, MSCs were cultured with HD serum or AS serum for 2 days and seeded in culture plates at a density of 2 × 10^4^ MSCs per well. After being cultured at 37 °C with 5% CO_2_ overnight, the cells were washed twice with freshly prepared assay medium (10 mM glucose, 1 mM pyruvate, and 2 mM l-glutamine) and incubated in a low-CO_2_ incubator. Meanwhile, three compounds (1 µM oligomycin, 2 µM FCCP, and 0.5 µM antimycin A and rotenone) were added into specific reagent ports in the probe plates. Finally, calibration and measurement were executed with the Seahorse XFe96 Analyzer.

### Mitochondrial ROS detection

Mitochondrial ROS levels were measured with MitoSOX^™^ Red Mitochondrial Superoxide Indicator (Invitrogen, M36008) according to the manufacturer’s instructions. Briefly, MSCs were cultured with HD serum or AS serum for 2 days and then incubated with MitoSOX (5 µM) diluted in Hanks’ Balanced Salt Solution (HBSS; Invitrogen, 14025092) for 15 min at 37 °C. Then, the cells were washed three times and stained with DAPI. The fluorescence of MitoSOX (excitation: 510 nm, emission: 580 nm) was determined immediately with a fluorescence microscope.

### Statistical analysis

All experiments were performed in triplicate, and MSC experiments were conducted with MSCs from three different donors. The investigator was blinded to the group allocation when assessing the outcome. All data were analyzed with SPSS 18.0 software and are presented as the mean ± standard deviation (SD). Difference comparisons between two groups were performed using independent-sample *t* tests and nonparametric tests. Correlation analyses were executed via Pearson correlation and linear regression analysis. Homogeneity test of variances and normal distribution were determined before each test. *p* < 0.05 was regarded as statistically significant.

## Results

### AS serum induced MSC senescence

**Fig. 1:**
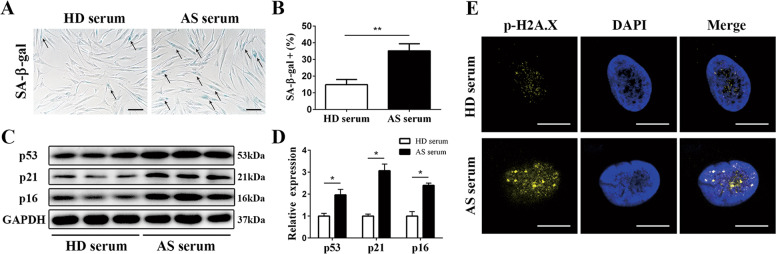
Compared with HD serum, AS serum facilitated MSCs senescence. **a** MSCs treated with AS serum displayed stronger SA-β-gal staining (black arrow) than those treated with HD serum (scale bar = 50 µm). **b** The percentages of SA-β-gal-positive cells among treated MSCs were 38.23 ± 7.47% and 15.66 ± 5.16% for AS serum and HD serum, respectively; *n* = 20, *p* < 0.001. **c**, **d** The expression of the cell cycle arrest-related proteins p53, p21, and p16 was detected by Western blotting, and the levels were higher in MSCs treated with AS serum than in those treated with HD serum. **e** The signal for p-H2A.X measured by immunofluorescence was more obvious in MSCs treated with AS serum than in those treated with HD serum (scale bar = 10 µm). The results are presented as the means ± SD (*n* = 20, determined by independent-sample *t* tests). All experiments were performed three independent times, *p < 0.05, **p < 0.01.

To determine the effect of AS serum on MSCs senescence, we performed SA-β-gal staining and found that the frequency of SA-β-gal-positive cells increased remarkably when cells were treated with AS serum (38.23 ± 7.47%, *p* < 0.001, *n* = 20) compared with HD serum (15.66 ± 5.16%) (Fig. 1a, b). In addition, we examined the expression of the cell cycle arrest-related proteins p53, p21, and p16. The results showed that the expression of all three cell cycle arrest-related proteins in MSCs was higher after treatment with AS serum than after treatment with HD serum (Fig. 1c, d). We also evaluated the phosphorylation level of H2A.X (p-H2A.X), a marker of DNA damage that usually accompanies cellular senescence^25^, and discovered that the fluorescence signal for p-H2A.X was more obvious in MSCs cultured with AS serum than in those cultured with HD serum (Fig. 1e). Furthermore, we analyzed the mRNA expression levels of SASP-related cytokines, including IL-6, IL-8, MCP1, MCP2, GRO, MIF, GM-CSF and three major immunoregulatory factors, IDO, LIF and TGF-β, in MSCs. The results showed that the levels of SASP-related cytokines were enhanced, while the expression levels of immunoregulatory factors were decreased in MSCs cultured with AS serum (Fig. S1A, B). Taken together, these findings demonstrated that in contrast to HD serum, AS serum dramatically facilitated the senescence of MSCs.

### MSCs located in the articular soft tissue of AS patients displayed distinct characteristics of senescence

**Fig. 2:**
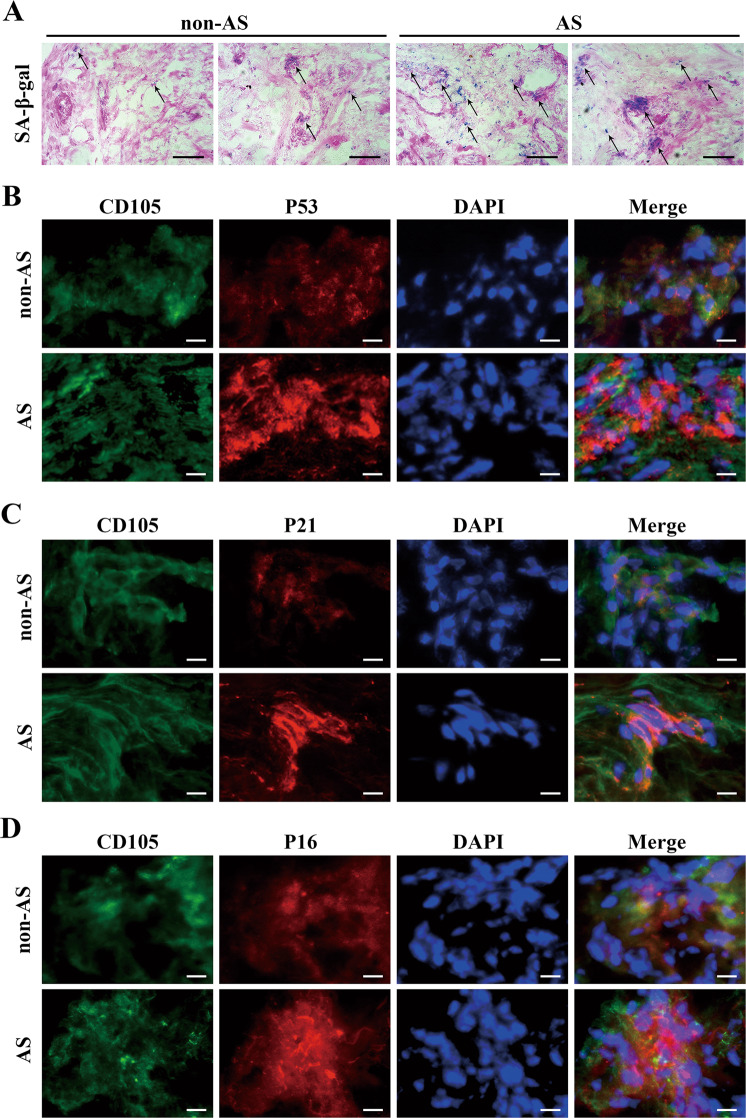
MSCs located in the articular soft tissue of AS patients displayed remarkable characteristics of senescence. **a** Tissues from AS patients displayed stronger SA-β-gal staining (black arrow) than those from non-AS donors (scale bar = 100 µm). **b**–**d** Immunofluorescence detection indicated increased levels of p53 (**b**), p21 (**c**), and p16 (**d**) in MSCs in articular soft tissue samples from AS patients than in those from non-AS patients (scale bar = 40 µm). All experiments were performed three independent times (*n* = 4).

To determine whether MSC senescence exists in vivo in AS patients, we collected articular soft tissue samples from four AS patients and four non-AS patients and performed SA-β-gal staining and immunofluorescence on frozen sections of these tissue samples. As shown in Fig. 2a, tissues from AS patients displayed stronger SA-β-gal staining than those from non-AS donors. In addition, in Fig. 2b–d, tissue-located MSCs in AS patients, which were labeled with CD105, expressed the cell cycle arrest-related proteins p53, p21, and p16 in greater abundance than tissue-located MSCs in non-AS patients, which indicated that MSC senescence was present to a larger extent in the AS patients.

### AOPPs were enhanced in AS serum and positively correlated with the senescence-promoting effect of AS serum

To explore the factors in AS serum responsible for MSC senescence, we first performed a cytokine array on HD and AS sera and found that CHI3L1 and PDGFA expression levels were increased in AS serum, which were further verified by ELISA (Fig. S2A). We further measured the effects of CHI3L1 and PDGFA on MSC senescence but found that neither CHI3L1 nor PDGFA influenced the SA-β-gal staining or the expression levels of p53, p21, and p16 in MSCs (Fig. S2B, C).

**Fig. 3:**
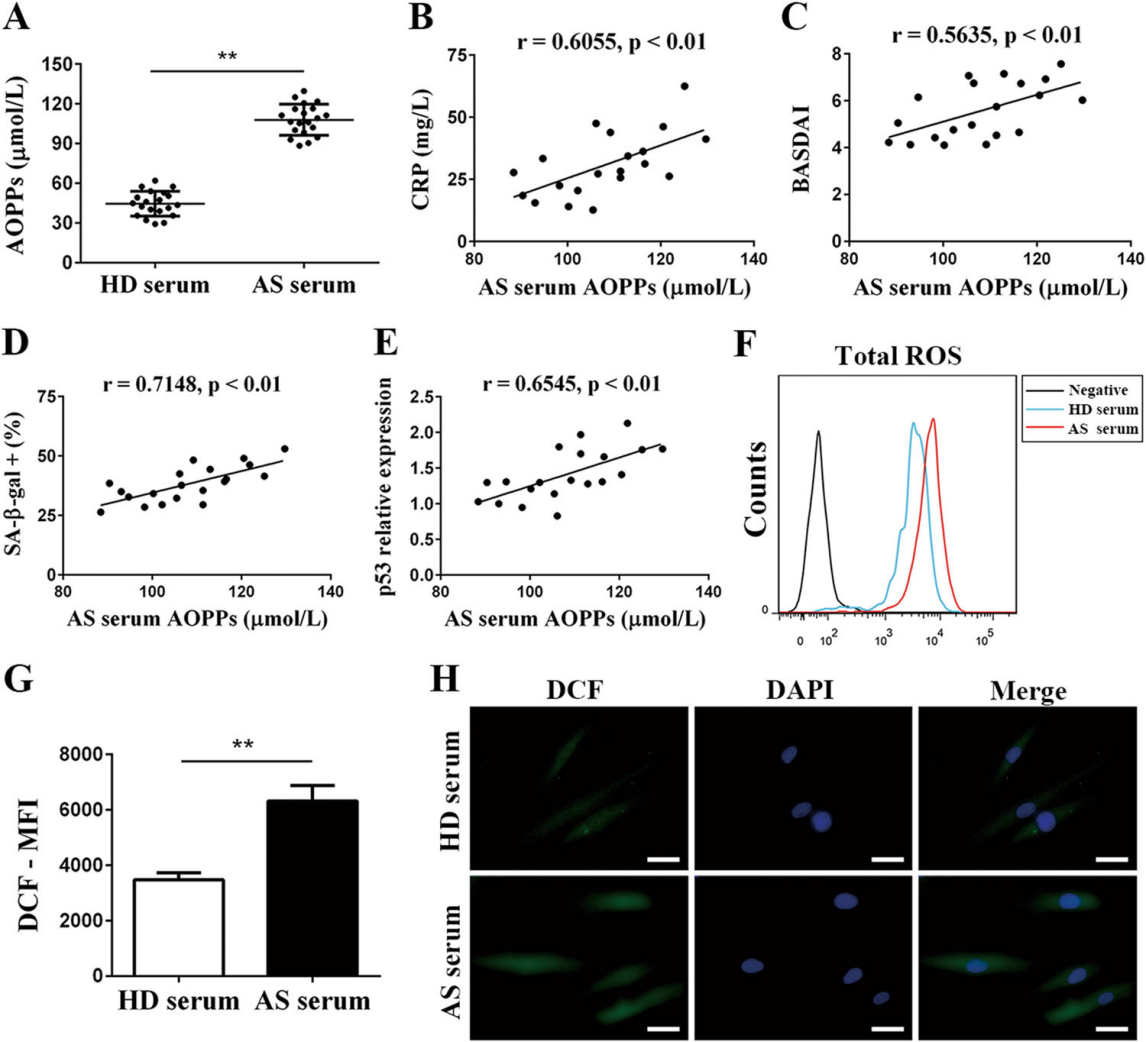
AS serum contained elevated AOPPs levels and could stimulate ROS production in MSCs. **a** The levels of AOPPs were higher in AS serum (108.0 ± 11.69 µmol/L) than in HD serum (44.63 ± 9.41 µmol/L); *p* < 0.001, *n* = 20. **b**–**e** The levels of AOPPs in AS serum were positively correlated with the level of CRP (*r* = 0.6055, *p* < 0.01) (**b**), BASDAI scores (*r* = 0.5635, *p* < 0.01) (**c**), the percentage of SA-β-gal-positive cells after treatment with AS serum (*r* = 0.7148, *p* < 0.01) (**d**), and the expression of the protein p53 in MSCs treated with AS serum (*r* = 0.65458, *p* < 0.01) (**e**–**g**). Flow cytometry detection revealed that the total ROS level was enhanced in MSCs treated with AS serum compared with those treated with HD serum. **h** AS serum-treated MSCs displayed a stronger signal for ROS than HD serum-treated MSCs under a fluorescence microscope (scale bar = 20 µm). The results in **a**, **f**, and **g** are presented as the means ± SD (*n* = 20, determined by independent-sample *t* tests). The data in **b**–**e** were determined by Pearson correlation and linear regression analysis (*n* = 20). All experiments were performed three independent times, *p < 0.05, **p < 0.01.

Oxidative stress is a major inducer of senescence in organisms^26^, and AOPPs are capable of inducing ROS production and subsequent cell cycle arrest^24^. Therefore, we suspected that AOPPs had a role in the senescence-promoting effect of AS serum. We detected the level of AOPPs and found that AOPPs expression was markedly elevated in AS serum (108.0 ± 11.69 µmol/L) compared with HD serum (44.63 ± 9.41 µmol/L, *p* < 0.001, *n* = 20) (Fig. 3a). Furthermore, we performed correlation analyses of the AOPPs level with the CRP level and disease activity. As expected, the level of AOPPs in AS serum was strongly and positively correlated with the CRP level and BASDAI scores (Fig. 3b, c). More critically, we determined that the AOPPs level possessed significant positive relations with the percentage of SA-β-gal-positive cells (Fig. 3d) and the expression of p53 (Fig. 3e), p21 and p16 (data not shown). In sum, these results suggested that the enhanced level of AOPPs was responsible for the senescence-promoting effect of AS serum.

### AS serum caused ROS excess in MSCs

Many studies have reported that ROS can be induced by AOPPs and play a vital role in triggering cellular senescence^24,27^. We wondered whether exposure to AS serum would increase ROS production in MSCs. Thus, we detected the ROS level with the probe DCFH-DA and discovered that the ROS levels of MSCs were quite higher in the AS serum group than in the HD serum group, as displayed by a stronger MFI for DCF measured by flow cytometry (Fig. 3f, g) together with brighter fluorescence found by fluorescence microscopy (Fig. 3h).

### N-acetylcysteine inhibited ROS production and rescued MSCs from senescence

**Fig. 4:**
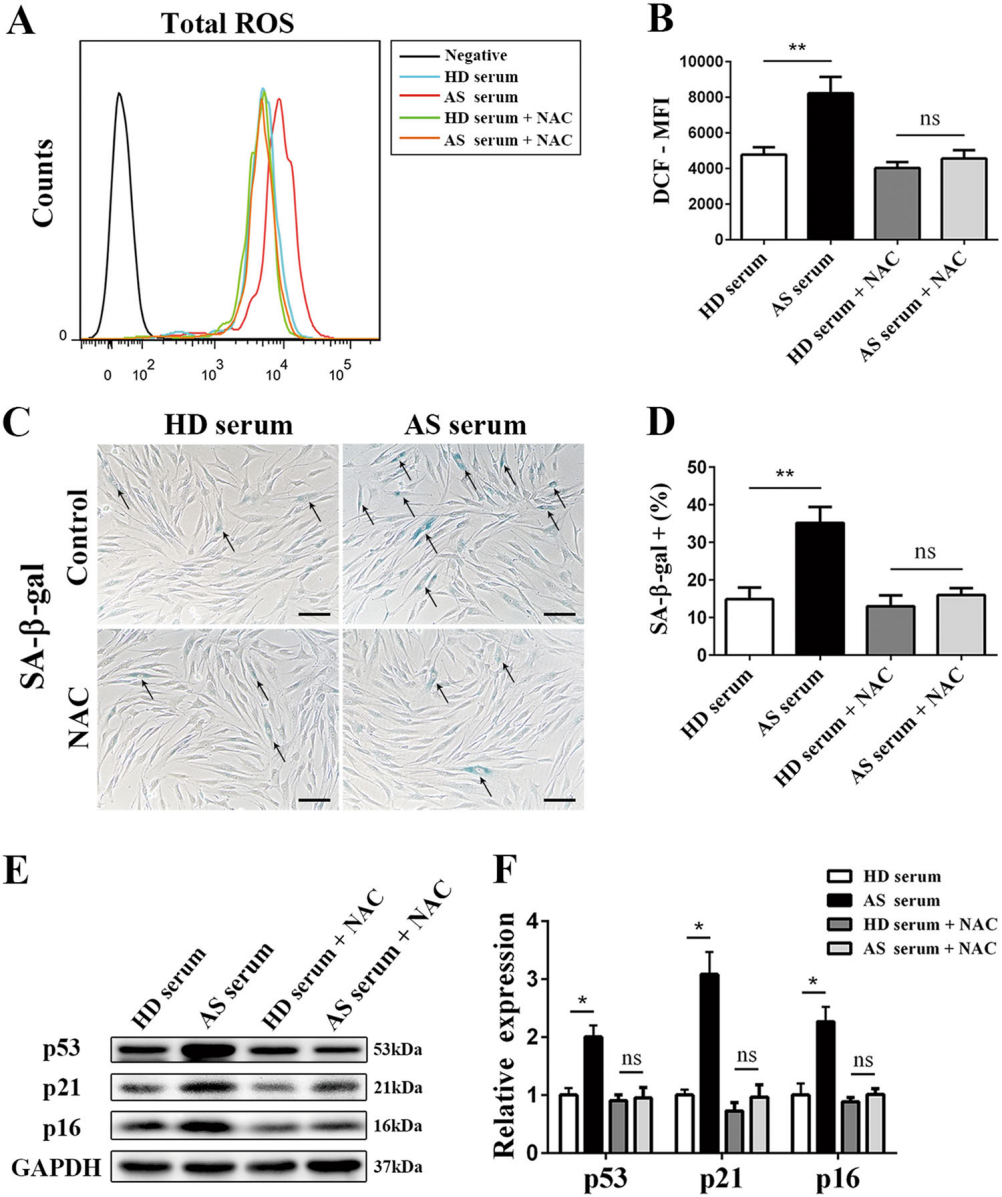
ROS inhibition via NAC rescued MSCs from the senescence caused by AS serum. **a**, **b** NAC reduced the ROS level in AS serum-treated MSCs to the level in HD serum-treated MSCs. **c**, **d** NAC rescued the extent of SA-β-gal staining of MSCs (black arrow) induced by AS serum (scale bar = 50 µm). **e**, **f** NAC decreased the expression levels of the cell cycle arrest-related proteins p53, p21, and p16 in AS serum-treated MSCs to the levels in HD serum-treated MSCs. The results are presented as the means ± SD (*n* = 10, determined by independent-sample *t* tests). All experiments were performed three independent times, *p < 0.05, **p < 0.01.

To validate the role of ROS in the senescence-promoting effect of AS serum, we suppressed the production of ROS with N-acetylcysteine (NAC), a widely used inhibitor of ROS^28^. As the level of ROS was reduced by NAC treatment (Fig. 4a, b), the increased percentage of SA-β-gal-positive cells (Fig. 4c, d) together with the expression of p53, p21, and p16 (Fig. 4e, f) in MSCs caused by AS serum returned to normal levels. These findings revealed that AS serum stimulated ROS production in MSCs and that NAC was able to reduce the ROS level and rescue MSC senescence induction by AS serum.

### Mitochondrial dysfunction of MSCs was mediated by AS serum

**Fig. 5:**
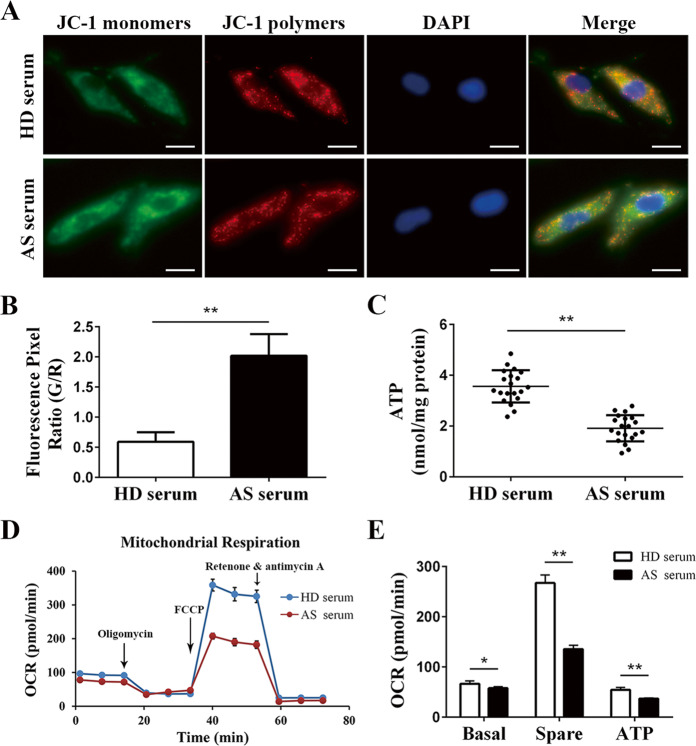
AS serum-mediated mitochondrial dysfunction in MSCs. **a** The mitochondrial membrane potential detected by the probe JC-1 showed reduced fluorescence of JC-1 polymers and elevated fluorescence of JC-1 monomers in MSCs treated with AS serum, which represented a reduced ∆Ψm in the AS serum-treated MSCs (scale bar = 10 µm). **b** The fluorescence index analyzed by ImageJ showed that the ratio of JC-1 monomers (green) to polymers (red) was higher in MSCs treated with AS serum than in those treated with HD serum, which implied a reduced ∆Ψm in the AS serum-treated MSCs. **c** ATP detection showed that AS serum treatment obviously reduced the ATP production of MSCs. **d**, **e** Mitochondrial oxygen consumption rate detection revealed that compared to HD serum, AS serum impaired the basal respiration, spare respiration and ATP production of MSCs. The results are presented as the means ± SD (*n* = 10, determined by independent-sample *t* tests). All experiments were performed three independent times, *p < 0.05, **p < 0.01.

The mitochondria are the major origin of cellular ROS^29^. To determine the mechanism responsible for the ROS excess, we determined the mitochondrial function of MSCs treated with AS serum or HD serum. First, we conducted ∆Ψm detection basing on JC-1 and found that compared to HD serum, AS serum obviously decreased the ∆Ψm of MSCs (Fig. 5a, b). In addition, the ATP production of MSCs was also reduced after AS serum treatment (Fig. 5c). Then, we determined the OCR and found that both the basal respiration and the spare respiration of MSCs were impaired by AS serum (Fig. 5d, e). In addition, reduced ATP production was also found in the OCR assay (Fig. 5d, e). Furthermore, we treated the MSCs with ROS scavenger NAC and measured the OCR and ATP production. The results showed that NAC treatment improved the OCR and ATP production in MSCs cultured with AS serum but not HD serum (Fig. S3A–C). In addition, with NAC treatment, there were not statistically significant differences in OCR and ATP production between MSCs treated with AS serum and MSCs treated with HD serum (Fig. S3A–C). In summary, all these results illustrated that AS serum treatment resulted in MSCs mitochondrial dysfunction.

### MQ reversed the excess mitochondrial ROS production and MSCs senescence induced by AS serum

**Fig. 6:**
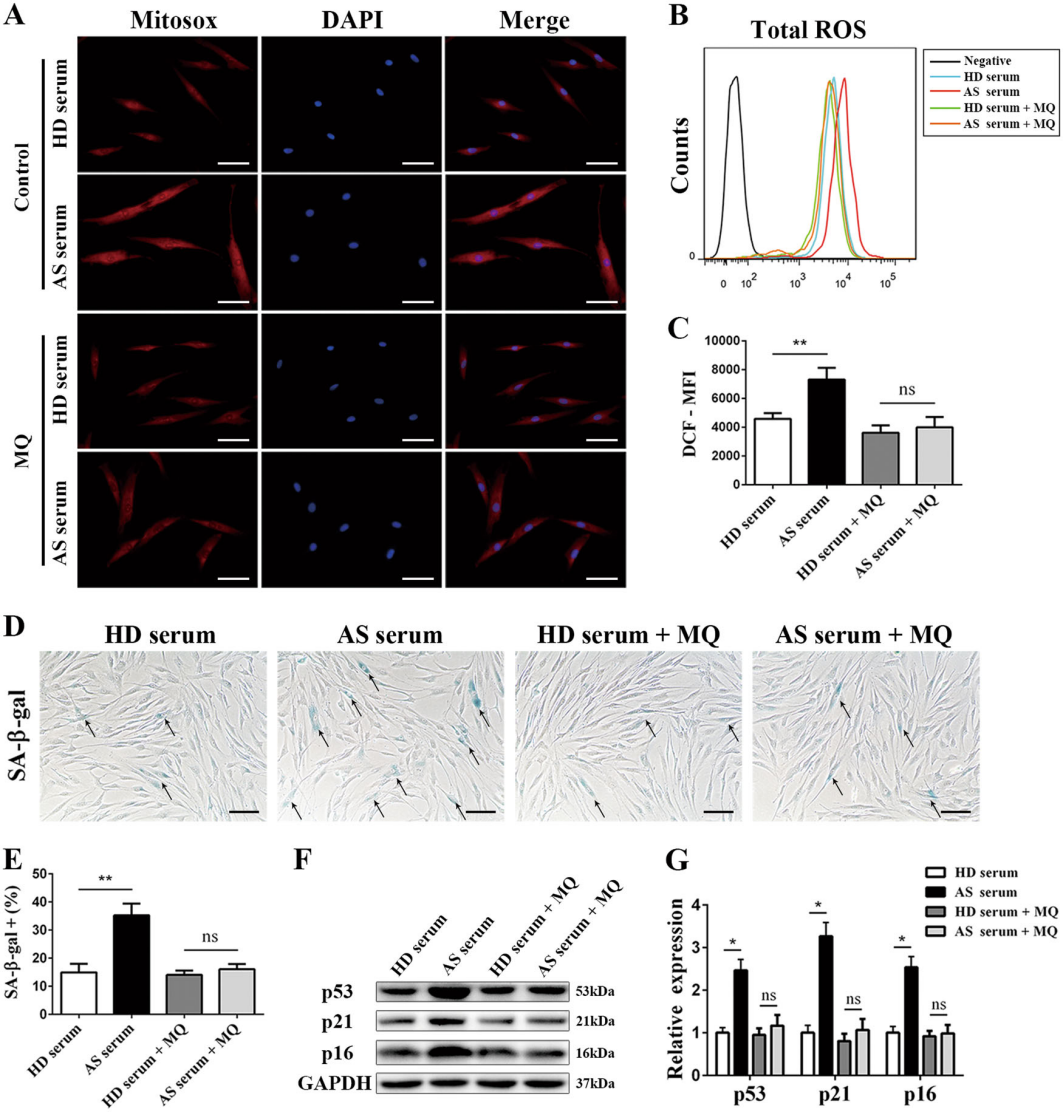
Mitoquinone decreased the ROS level and reversed the senescence of MSCs caused by AS serum. **a** Mitochondrial ROS evaluation with MitoSOX showed that AS serum increased the mitochondrial ROS levels of MSCs and that MQ pretreatment eliminated this effect (scale bar = 40 µm). **b**, **c** The total ROS level detected by the probe DCFH-DA showed that MQ reduced the total ROS level of AS serum-treated MSCs to the level of HD serum-treated MSCs. **d**, **e** The SA-β-gal staining assay indicated that MQ decreased the percentage of SA-β-gal-positive MSCs (black arrow) treated with AS serum (scale bar = 50 µm). **f**, **g** Western blot analysis revealed that MQ reversed the expression of the cell cycle arrest-related proteins p53, p21, and p16 in MSCs treated with AS serum. The results are presented as the means ± SD (*n* = 10, determined by independent-sample *t* tests). All experiments were performed three independent times, *p < 0.05, **p < 0.01.

To determine whether mitochondrial dysfunction was the source of the excess ROS, we detected mitochondrial ROS by using the probe MitoSOX, and the results showed that in comparison with HD serum, AS serum enhanced the mitochondrial ROS level of MSCs (Fig. 6a). In addition, by adding mitoquinone (MQ), a mitochondrion-targeted antioxidant^30^, the mitochondrial ROS level (Fig. 6a) together with the total ROS level (Fig. 6b, c) of MSCs treated with AS serum returned to the levels seen in HD serum-treated MSCs. Furthermore, MQ pretreatment successfully alleviated the influence of AS serum on MSCs senescence, which was indicated by the reduced percentage of SA-β-gal-positive cells (Fig. 6d, e) and expression of p53, p21, and p16 (Fig. 6f, g). Taken together, these data confirmed that AS serum increased mitochondrial ROS levels in MSCs and that targeting mitochondrial ROS was able to relieve the senescence-promoting effect of AS serum.

## Discussion

In this study, we focused on the influence of the AS serum environment on MSCs and discovered that compared to HD serum, AS serum strikingly facilitated MSCs senescence. Further mechanistic studies revealed that AOPPs in AS serum mediated mitochondrial dysfunction, inducing excess ROS production that resulted in MSC senescence.

The microenvironment is of great importance in organisms, and the serum environment is a vital part of it^31,32^. Many studies have reported that an abnormal serum environment affects cell functions and participates in disease development^19,33^. For AS, studies have found that the levels of various inflammatory factors such as TNF-α and IL17 and oxidative stress are abnormally increased in AS serum^3,34^. However, the specific influence of AS serum on cells in organism has rarely been reported. MSCs possess a strong immunomodulatory ability and the capacity for trilineage differentiation^6^. MSC dysfunction participates in several inflammatory and articular diseases^7^. Previously, we found that MSCs from AS patients displayed multiple functional abnormalities^9–11^; however, the underlying reason was uncertain but might be related to the AS serum environment. Therefore, in this study, we explored the impact of AS serum on MSCs and confirmed that in contrast to HD serum, AS serum dramatically facilitated MSCs senescence. In addition, we discovered enhanced expression of the cell cycle arrest-related proteins p53 and p21 in MSCs from AS articular tissue samples by immunofluorescence, which was in agreement with our in vitro findings. Similar to what we found in AS serum, serum from SLE and SSc patients also accelerates the senescence of MSCs, thereby influencing disease development^19,20^. These findings together implied pathogenic roles for the morbid serum environment and MSCs senescence in autoimmune diseases. Accordingly, we proposed that the chronically morbid serum microenvironment of AS participates in disease development through facilitating MSCs senescence.

Though serum from several different autoimmune disease patients have similar impacts on MSCs senescence, the intrinsic mechanisms are different. In SLE, increased leptin and NAP2 levels are responsible for the senescence-promoting effect on MSCs^19^. In SSc, serum-derived oxidative stress facilitates MSCs senescence^20^. Previously, we discovered an imbalance between oxidant biomarker and antioxidant levels in AS serum, which implied that disordered redox may participate in AS^5^. In this study, we found that AOPPs levels in AS serum were strongly and positively correlated with the extent of SA-β-gal staining and the expression of cell cycle arrest-related proteins in MSCs cultured with AS serum. In AS patients, serum AOPPs levels have been reported to be elevated and positively corelated with the CRP level, the erythrocyte sedimentation rate and disease activity indexes, such as the BASDAI^35^, which agreed with the results of our study. Then, whether AOPPs are simply an accompanying marker or are actually engaged in AS pathogenesis remains to be determined. Previously, studies illustrated that AOPPs were closely related to inflammation and immune dysregulation^36^. In addition, recent studies have reported that AOPPs are not only an oxidant marker but also capable of inducing oxidative stress and cell cycle arrest^24^. In view of these reports and our findings reported in this study, we speculate that AOPPs are not only an accompanying marker but also participants in AS pathogenesis through the induction of MSCs senescence and may be a potential therapeutic target for AS treatment.

Excess ROS is a common cause of cellular senescence and has been implicated in several inflammatory diseases^27,37^. In AS, studies have reported that ROS levels are enhanced in leukocytes and ROS are considered to be a potential mediator of AS pathogenesis^38,39^. We discovered that AS serum stimulated ROS production in MSCs and targeting ROS was able to rescue MSCs from senescence. Previous studies discovered that senescent MSCs exhibited impaired immunoregulatory function and elevated pro-inflammatory factor expression, which might cause an inflammatory reaction^19^. In addition, an inflammatory stimulus was able to initiate a respiratory burst and result in oxidative stress production^40^. Furthermore, it has been reported that oxidative stress can serve as a primary disorder and induce a secondary disorder, which further increases oxidative stress^41^. All these findings imply that such a vicious cycle might occurs in AS: the oxidative stress in AS serum induce excessive ROS production and MSCs senescence, and then the senescent MSCs lead to an inflammatory reaction, with the consequent inflammation further increasing oxidative stress. Therefore, we propose that ROS may be of great importance in AS development, especially in chronic inflammation, and that ROS are a potential target for AS therapy. Consistent with our assumption, previous studies have reported that several kinds of drugs for AS, including nonsteroidal anti-inflammatory drugs (NSAIDs) and sulfasalazine, are capable of inhibiting ROS^42,43^, which may contribute to their anti-inflammatory effects. Further clinical studies targeting ROS are required to validate these hypotheses.

Mitochondria are the major origin of ROS, and mitochondrial dysfunction participates in ageing and several autoimmune diseases, such as RA and inflammatory bowel disease^44,45^. However, few studies have explored the role of mitochondria in AS. There are only analytical studies that have predicted that the pathway of mitochondrial dysfunction and some mitochondrion-related proteins may be involved with AS^46,47^. In this study, we explored mitochondrial function in AS and discovered that the AS serum environment induced mitochondrial dysfunction in MSCs and subsequently raised ROS levels and caused MSCs senescence. Thus, we propose that mitochondrial dysfunction participates in AS pathogenesis and may be a new therapeutic target in AS. Recent studies have reported that the JAK/STAT blockers tofacitinib and filgotinib, which are capable of improving mitochondrial function, are efficacious in the treatment of AS^48,49^, supporting a role for the mitochondria in AS.

Then, what is the specific mechanism underlying the mitochondrial dysfunction caused by AS serum? The electron transfer chain (ETC), which mainly consists of four components named complexes I to IV, is the core part of the mitochondria^50^. It can be disrupted by oxidative stress, resulting in a reduced oxidative phosphorylation rate and decreased ATP production, followed by ROS overproduction^51^. Our results showed that basal respiration and spare respiration together with ATP production were impaired in the AS serum environment, indicating impairment of the ETC. A genes analysis study of AS revealed that the most significant gene ontology (GO) enrichment was in the category of respiratory electron transport chain, which also suggested the presence of ETC impairment in AS^46^. Furthermore, this study also reported that several complex I- and IV-related genes exhibited downregulated expression in AS, implying the mechanism of ETC impairment in AS. Further studies are supposed to explore the specific mechanism underlying ETC impairment and therapies targeting mitochondrial dysfunction in AS.

MSC dysfunction is closely related to inflammation in organisms and is implicated in autoimmune diseases, including RA, SLE, and AS^52^. Recently, studies have found that cellular senescence influences both the immunoregulation and differentiation abilities of MSCs and, therefore, mediates disease development^18,19,53^. We found that the AS serum environment induced MSC senescence, but how senescent MSCs participate in AS is still being evaluated. Chronic inflammation is the core part of AS pathogenesis, and the dysregulation of macrophages and T cells plays a crucial role in this process^2^. In AS, T cells and macrophages are the most frequent cells located in early and active sacroiliitis^54^. In addition, the pro-inflammatory M1 macrophages, Th1 cells and Th17 cells are expanded in AS and lead to the abnormally elevated levels of TNF-α and IL-17, two factors mainly responsible for the chronic inflammation and joint damage in AS^55^. Under normal conditions, MSCs possess strong immunomodulatory capability on macrophages and T cells. MSCs are capable of shifting pro-inflammatory M1 macrophages into anti-inflammatory M2 macrophages by secreting immunoregulatory factors^56^. In addition, MSCs are able to modulate the Th1/Th2 and Th17/Treg balance, thereby reducing the production levels of pro-inflammatory cytokines such as TNF-α and IL-17^57,58^. However, when entering senescence, MSCs immunomodulatory ability becomes impaired, and a pro-inflammatory SASP occurs^19^. In this study, we found that AS serum-induced senescent MSCs expressed reduced levels of immunomodulatory factors, including LIF, IDO and TGF-β, indicating that the suppressive effects of MSCs on T cells and macrophages become impaired and that serious pro-inflammatory phenotypes of T cells and macrophages may occur in AS. In addition, we also revealed that senescent MSCs induced by AS serum possessed an SASP with higher expression of several pro-inflammatory cytokines, such as IL-6, MIF, MCP1, and GM-CSF. These cytokines are closely related to AS pathogenesis. IL-6 and MIF are increased in AS and capable of activating T cells and macrophages to express TNF-α or IL-17^59,60^. MCP1 and GM-CSF play important roles in the pro-inflammatory function and migration of macrophages^61,62^. Taken together, we deem that senescence establishment in MSCs could lead to pro-inflammatory phenotypes of T cells, macrophages and excessive production of TNF-α and IL-17, thereby triggering or aggravating the inflammation in AS.

In past decades, MSCs have been widely used to treat inflammatory diseases^63^. While many clinical studies have reported that the efficacy of MSCs infusion are supposed to be improved. For instance, Sun reported that MSCs transplantation was a hopeful treatment for SLE but only 32.5% of patients achieved a major clinical response^64^. Previously, we determined that intravenous infusion of MSCs from HDs was effective for AS treatment, but a considerable proportion of the patients did not respond^12^. We speculated that this treatment failure might be related to the impact of the patients’ internal microenvironment on the functions of MSCs. In this study, we discovered that the AS serum environment strikingly facilitated the senescence of MSCs. Likewise, the infused MSCs would be affected similarly, thereby losing their immunomodulatory function and therapeutic effect. These findings implied that targeting oxidative stress in the serum might be a feasible way to improve the outcome of MSCs therapy in AS.

In conclusion, we demonstrated that the AS serum environment mediated mitochondrial dysfunction and facilitated MSCs senescence. These findings may help elucidate the pathogenesis of AS and provide new targets for AS treatment. However, there are some limitations to this study. What is the specific mechanism of mitochondrial dysfunction? What are the efficacies of therapies aimed at the abovementioned targets? Further mechanistic and clinical studies are required to solve these problems.

## Supplementary information

Figure S1

Figure S2

Figure S3

Table S1

Supplementary Figure Legends
