# Supplementary figures and images for: Oxidative stress-mediated mitochondrial dysfunction facilitates mesenchymal stem cell senescence in ankylosing spondylitis
Source: Cell Death Dis. 2020 Sep 17;11(9):775. doi: 10.1038/s41419-020-02993-x (PMC7498590; doi:10.1038/s41419-020-02993-x)

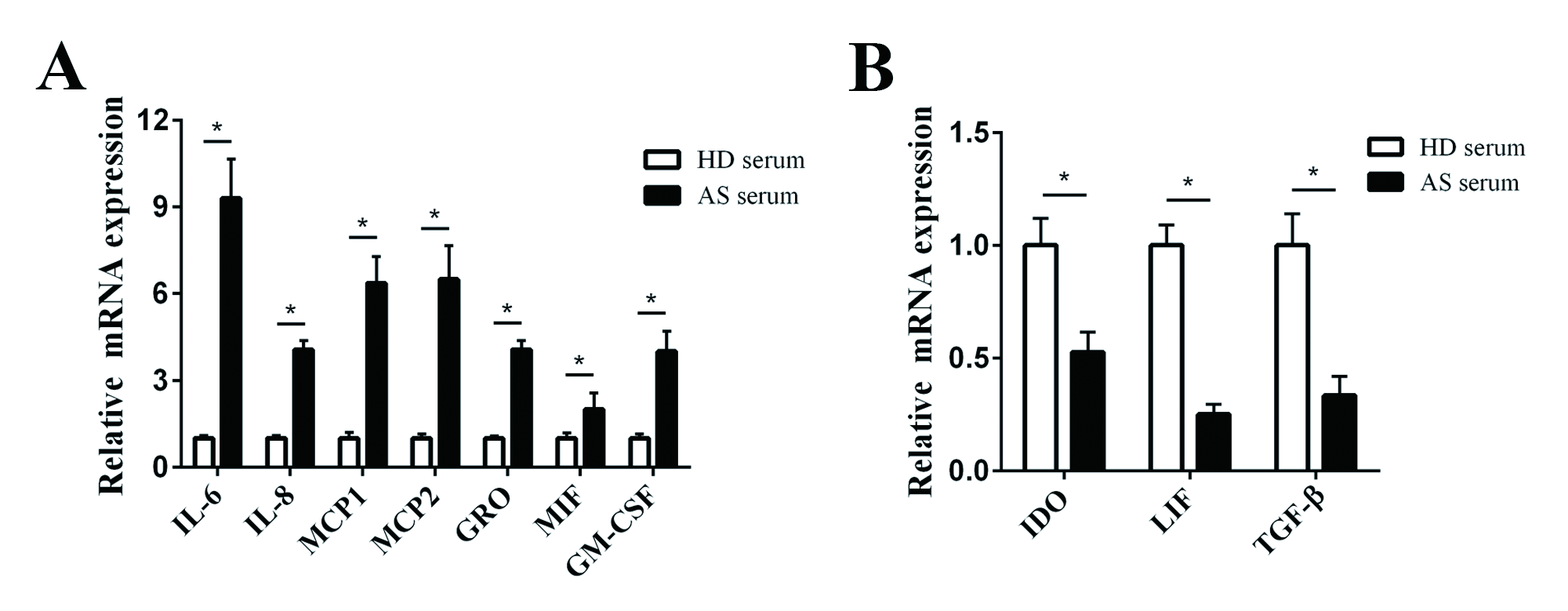

Supplement: Supplementary file 1 — Figure S1 [file 41419_2020_2993_MOESM1_ESM.tif]

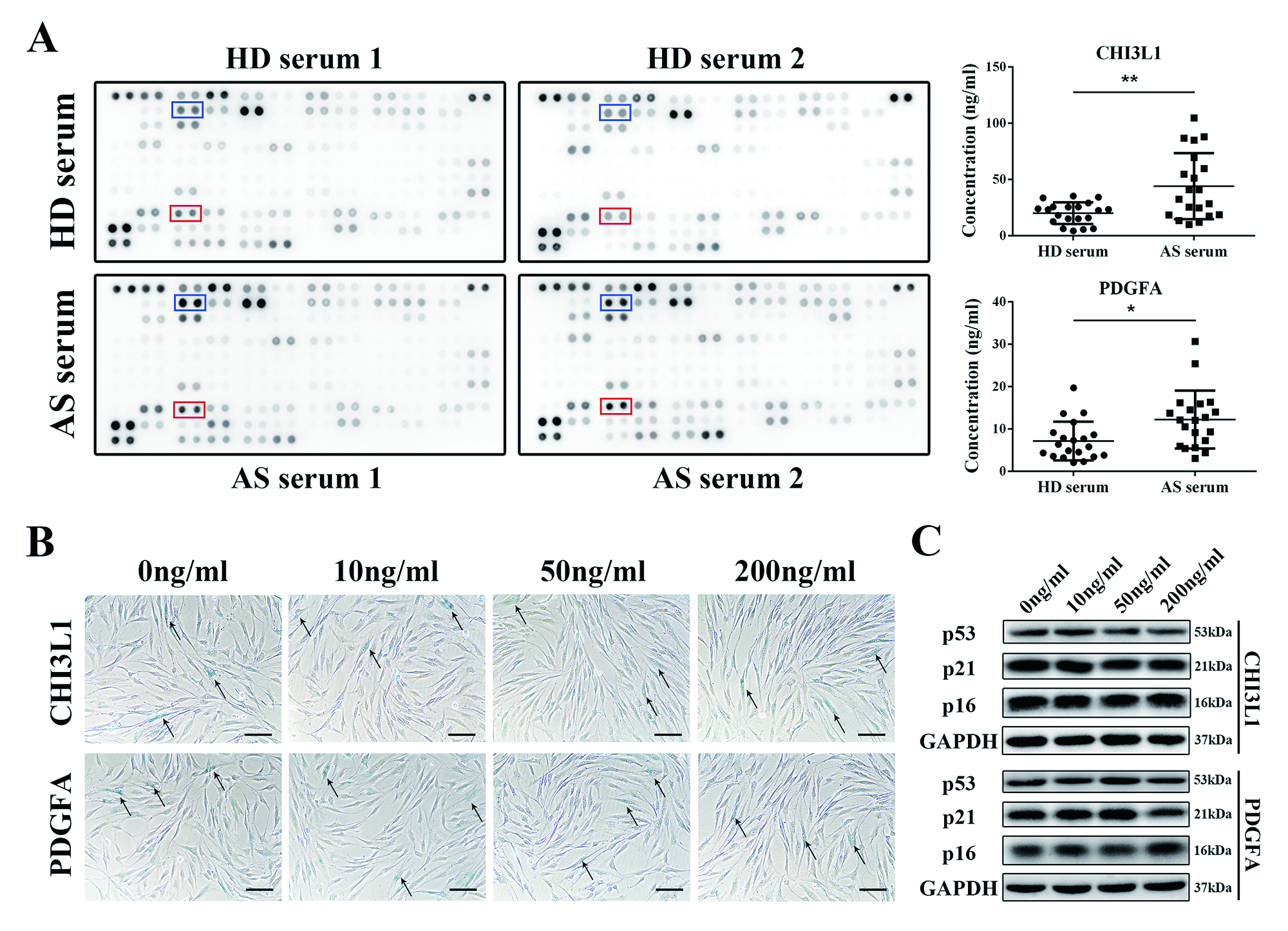

Supplement: Supplementary file 2 — Figure S2 [file 41419_2020_2993_MOESM2_ESM.tif]

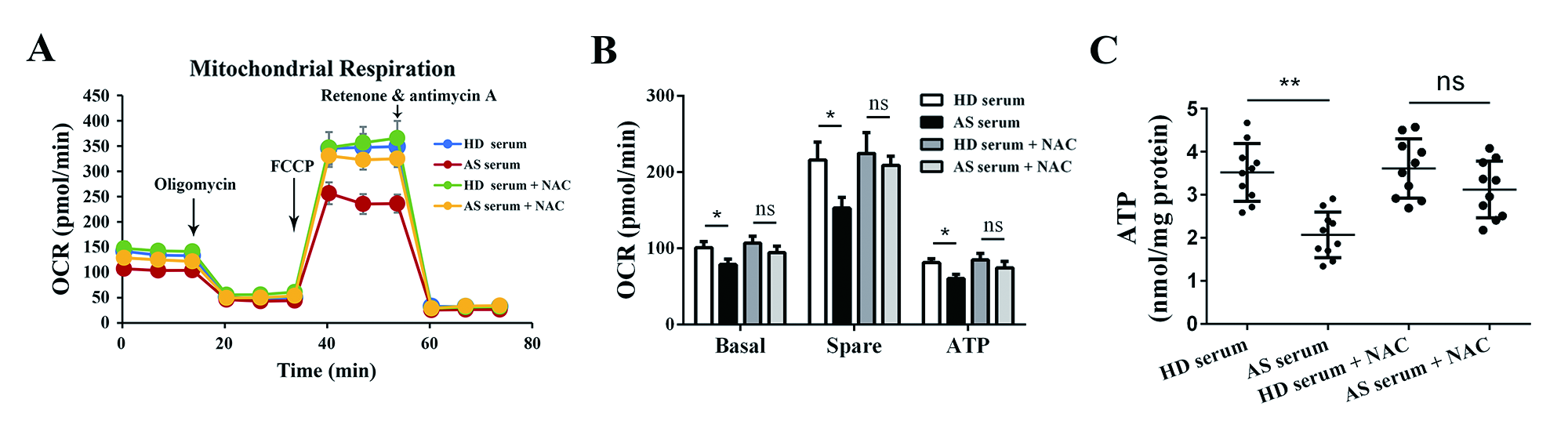

Supplement: Supplementary file 3 — Figure S3 [file 41419_2020_2993_MOESM3_ESM.tif]
