# Supplementary material for: Oxidative stress-mediated mitochondrial dysfunction facilitates mesenchymal stem cell senescence in ankylosing spondylitis
Source: Cell Death Dis. 2020 Sep 17;11(9):775. doi: 10.1038/s41419-020-02993-x (PMC7498590; doi:10.1038/s41419-020-02993-x)
Supplement: Supplementary file 4 — Table S1 [file 41419_2020_2993_MOESM4_ESM.docx]

| Characteristics | AS patients | Healthy donors | p-value |
| --- | --- | --- | --- |
| Numbers | 20 | 20 | NS |
| Age, year | 29.3 ± 7.6 | 26.6 ± 5.9 | NS |
| Sexuality | Male: 12, Female: 8 | Male: 10, Female: 10 | NS |
| Disease duration, year | 5.53 ± 3.84 | - | - |
| CRP, mg/L | 30.8 ± 12.8 | 3.23 ± 1.47 | < 0.01 |
| ESR, mm/h | 40.7 ± 15.1 | 9.03 ± 3.71 | < 0.01 |
| BASDAI | 5.57 ± 1.19 | - | - |
| ASDAS | 4.24 ± 1.53 | - | - |

**Table S1:** The characteristics of the study subjects

Abbreviations: AS, ankylosing spondylitis; CRP, C-reactive protein; ESR, erythrocyte sedimentation rate; BASDAI, Bath Ankylosing Spondylitis Disease Activity Index; ASDAS, Ankylosing Spondylitis Disease Activity Score; NS, nonsense. All data were presented as mean ± standard deviation (SD).
