# Supplementary material for: Oxidative stress-mediated mitochondrial dysfunction facilitates mesenchymal stem cell senescence in ankylosing spondylitis
Source: Cell Death Dis. 2020 Sep 17;11(9):775. doi: 10.1038/s41419-020-02993-x (PMC7498590; doi:10.1038/s41419-020-02993-x)
Supplement: Supplementary file 5 — Supplementary Figure Legends [file 41419_2020_2993_MOESM5_ESM.docx]

**Supplementary Figure Legends**

**Figure S1.** AS serum-treated MSCs expressed enhanced levels of SASP-related cytokines but decreased levels of immunomodulatory factors. **A**. The mRNA expression measured by qPCR revealed that AS serum-treated MSCs possessed higher levels of IL-6, IL-8, MCP1, MCP2, GRO, MIF and GM-CSF compared with MSCs treated with HD serum. **B**. The mRNA expression measured by qPCR showed that AS serum-treated MSCs possessed reduced levels of IDO, LIF and TGF-β compared with MSCs treated with HD serum. The results are presented as the means ± SD (n = 10, determined by independent-sample t-tests). All experiments were performed three independent times, *P < 0.05.

**Figure S2.** CHI3L1 and PDGFA were increased in AS serum but did not influence MSC senescence. **A**. A cytokine array was performed on HD and AS sera and implied that two cytokines, CHI3L1 and PDGFA, were increased in AS serum compared to HD serum. ELISA experiments also showed that these two cytokines were enhanced in AS serum (n=20, determined by independent-sample t-tests, *P < 0.05, **P < 0.01.). **B.** The SA-β-gal staining showed that neither CHI3L1 nor PDGFA influenced the SA-β-gal staining of MSCs (n = 10, determined by independent-sample t-tests, scale bar = 50 µm). **C**. The protein levels of p53, p21 and p16 in MSCs were not influenced by CHI3L1 or PDGFA (n = 10, determined by independent-sample t-tests). All experiments were performed three independent times.

**Figure S3.** NAC improved the OCR and ATP production levels in MSCs cultured with AS serum. **A** and **B**. Mitochondrial oxygen consumption rate detection revealed that NAC treatment improved the OCR and ATP production in AS serum-treated MSCs but not HD serum-treated MSCs. With NAC treatment, the differences in OCR and ATP production between MSCs treated with AS serum and MSCs treated with HD serum became reversed. **C**. ATP detection revealed that NAC treatment improved the ATP production in AS serum-treated MSCs but not HD serum-treated MSCs. With NAC treatment, the differences in ATP production between MSCs treated with AS serum and MSCs treated with HD serum became reversed. The results are presented as the means ± SD (n = 10, determined by independent-sample t-tests). All experiments were performed three independent times, *P < 0.05, **P < 0.01.
